# The Progress of Chitosan-Based Nanoparticles for Intravesical Bladder Cancer Treatment

**DOI:** 10.3390/pharmaceutics15010211

**Published:** 2023-01-07

**Authors:** Chong Yu, Shuai Wang, Wing-Fu Lai, Dahong Zhang

**Affiliations:** 1 Urology & Nephrology Center, Department of Urology, Zhejiang Provincial People’s Hospital, Affiliated People’s Hospital, Hangzhou Medical College, Hangzhou 310014, China; 2 Department of Applied Biology and Chemical Technology, Hong Kong Polytechnic University, Hong Kong, China

**Keywords:** chitosan, nanoparticle, bladder cancer, intravesical drug delivery

## Abstract

Bladder cancer (BC) is the most frequently occurring cancer of the urinary system, with non-muscle-invasive bladder cancer (NMIBC) accounting for 75–85% of all the bladder cancers. Patients with NMIBC have a good survival rate but are at high risk for tumor recurrence and disease progression. Intravesical instillation of antitumor agents is the standard treatment for NMIBC following transurethral resection of bladder tumors. Chemotherapeutic drugs are broadly employed for bladder cancer treatment, but have limited efficacy due to chemo-resistance and systemic toxicity. Additionally, the periodic voiding of bladder and low permeability of the bladder urothelium impair the retention of drugs, resulting in a weak antitumoral response. Chitosan is a non-toxic and biocompatible polymer which enables better penetration of specific drugs to the deeper cell layers of the bladder as a consequence of temporarily abolishing the barrier function of urothelium, thus offering multifaceted biomedical applications in urinary bladder epithelial. Nowadays, the rapid development of nanoparticles significantly improves the tumor therapy with enhanced drug transport. This review presents an overview on the state of chitosan-based nanoparticles in the field of intravesical bladder cancer treatment.

## 1. Introduction

In men, bladder cancer ranks fourth in terms of malignancies [1]. The costs associated with the surveillance and treatment of non-muscle-invasive bladder cancer (NMIBC) make bladder cancer one of the most expensive malignancies to be treated [2]. To prevent recurrence and progression of NMIBC, a majority of patients undergo repeated transurethral resections of the bladder tumor (TURBT) and intravesical drug delivery (IDD) [3]. Immunotherapy with bacillus Calmette–Guérin (BCG) is the most common intravesical treatment; nevertheless, intravesical chemotherapy using mitomycin C (MMC) or gemcitabine can be used both preoperatively and postoperatively [4].

However, a number of factors limit the effectiveness of intravesical chemotherapy. As urine cycles through the bladder continuously, chemotherapeutic agents are continuously washed out, limiting their contact time with the urothelium. Additionally, the bladder is composed of multiple layers, and the urothelium acts as a barrier to prevent intravesical agents from reaching deeper muscle layers [5]. Furthermore, the capillaries rapidly clear agents that penetrate the urothelium from the muscularis propria, resulting in systemic absorption and preventing adverse effects associated with their action on the bladder wall [6]. To address these shortcomings, new chemotherapeutic agents, treatment regimens, and drug delivery techniques are being developed.

Nowadays, bioadhesive colloidal delivery systems such as microspheres, microparticles, and nanoparticles (NPs) are promising delivery systems for intravesical chemotherapeutic agents [6]. In particular, NPs were found to improve drug penetration through urothelium and increase intravesical absorption of encapsulated drugs [7]. A number of NPs could achieve higher efficiency by interacting with mucus in an intimate manner due to their bioadhesive properties [8].

The mucoadhesive properties of chitosan make it a popular polymer for use in these treatment regimes [9]. The electrostatic interactions between chitosan and mucins in the mucus layer result in enhanced bioadhesion, making chitosan an ideal polymer for intravesical delivery [10]. Moreover, chitosan might promote structural reorganization of proteins associated with tight junctions, resulting in improved absorption of hydrophilic drugs [11,12].

Biomedical applications of chitosan-based nanomaterials have shown great success as antimicrobial agents and also act as carriers for drug delivery [13]. Moreover, chitosan derivatives as well as chitosan nanoparticles (ChNPs) have been extensively investigated for their potential applications in the field of biomedicine [14]. This review presents an overview on the state of chitosan-based nanoparticles in the field of intravesical bladder cancer treatment. The preparation, modification, and application of these drugs in bladder cancer therapeutics have received special attention.

## 2. Bladder Anatomy and Limitations of Drug Delivery

### 2.1. Bladder Physiology

Essentially, the urinary bladder is a hollow, spherical structure that stores urine for a short time. The average volume is approximately 400–600 mL and several factors affect the volume of urine in the bladder, including gender, ethnicity, and race. The first sensation of urination typically occurs when approximately 150 to 200 mL of urine is collected in the bladder. Detrusor muscles regulate urination through the myovesical plexus within the bladder, which sends the voiding signal to the bladder to control the amount and frequency of urination. 

Besides periodic voiding, the intrinsic bladder structure contributes an additional barrier to efficient drug absorption. The bladder wall is composed of multiple layers, including urothelium, lamina propria, detrusor muscles, and serosa. Between the blood and urine, urothelium, also known as the bladder permeability barrier (BPB), acts as an impermeable barrier [15]. While similar in function to the blood–brain barrier, the urothelium is required to be far more resistant to molecules as a storage organ. As the primary function of the BPB is to prevent the diffusion of toxic and pathogenic substances into the system circulation, it serves as a protective barrier. 

A specific protein array and tight junctions between umbrella cells are responsible for the barrier’s impermeability. Umbrella cells are impermeable because they have two distinct morphological characteristics. Firstly, in these cells, the apical membrane is covered with scallop-shaped plaques, separated by hinges of plasma membrane. Due to plaques on the outer leaflet of the apical membrane, the asymmetrical unit membrane (AUM) is formed with a thicker outer leaflet than inner leaflet [16]. In addition, the cells have a large number of cytoplasmic vesicles joined together by cytoskeletal fibrils which coalesce at the tight junctions and form desmosomes in the basal membrane [17].

### 2.2. Bladder Limitations for Intravesical Drug Delivery

**Figure 1:**
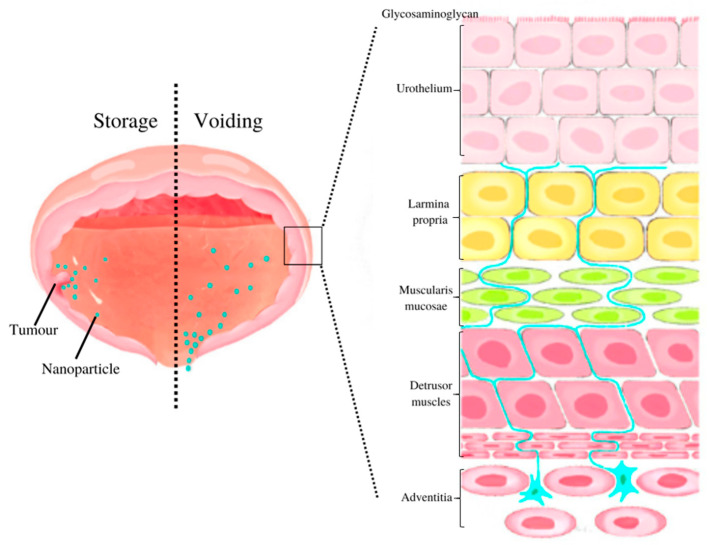
Bladder wall anatomy.

Clinically, the intravesical instillation of therapeutic agents is usually performed after surgical operation of TURBT. With current IDD, a large amount of the drug is infused directly into the bladder tumor site without increasing systemic blood levels; however, as shown in Figure 1, the drug is diluted eventually because of the constant urine production. As a result of periodical voiding, drugs infused into the body are washed out by the urine, thus resulting in a shortening of action duration and the need for frequent dosing, which increases the risk of systemic toxicity. The urothelial layer, however, prevents drugs from reaching the tissues even in diseased conditions. Additionally, the mucin layer comprises negatively charged glycosaminoglycans (GAGs), which adhere to the luminal side and augment the property of the barrier. As a result, foreign substances are prevented from adhering to the urothelial surface and solutions are prevented from penetrating the cell membranes.

### 2.3. Chitosan: An Ideal Candidate for Intravesical Drug Delivery

An optimized IDD system must increase the retention time of the drug in the bladder, while allowing the drug to penetrate the bladder wall for its local antitumor effect. Polysaccharides such as chitosan are frequently used for increasing drug permeability through the urothelium. Considering its biocompatible properties, biodegradability, bioactivity, polycationicity, and the presence of reactive alcohol and amine groups, chitosan is ideal for intravesical drug delivery [18]. The use of chitosan carriers for mitomycin C (MMC) on the bladder surface was studied by Eroglu et al. [19]. A bioadhesion test was conducted on the drug-loaded cylindrical chitosan carriers after they swelled in water. Furthermore, they kept the carriers in contact with a sheep bladder mounted on a platform and then slowly lowered the platform to remove the carrier from the bladder. According to the results, chitosan with a higher molecular weight has greater adhesive strength, suggesting that longer polymer chains provide a better interaction with mucous membrane GAGs. Additionally, greater cross-linking reduced the adhesive force because the cross-linker (glutaraldehyde) occupied more of the functional groups and reduced the interaction with the mucin layer. With chitosan of a higher molecular weight, drug MMC was also less released from carriers. Recently, in a study by Burjak et al. [20], Eudragit polymer microspheres were coated with chitosan, carboxymethylcellulose (CMC), and polycarbophil (PC) to study mucoadhesive properties. Microspheres increase the drug–tissue contact and prevent toxic effects. The PC microspheres showed maximum swelling, and therefore, the drug was released from the matrix the fastest. Compared with the other two polymers, chitosan showed an extremely high mucoadhesion on the bladder wall.

## 3. Preparation, Characterization, and Modification of ChNPs

As the main component of fungi and invertebrates, chitin is one of the most abundant polysaccharides in nature. Natural linear polysaccharide chitosan, obtained from alkaline hydrolysis of chitin, is cationic and hydrophilic. Chitosan is a non-toxic and biocompatible polymer with a random distribution of β-(1,4)-linked d-glucosamine (deacetylated) units and N-acetyl-d glucosamine units. The abundant hydroxyl (−OH) and amine (−NH_2_) functional groups of chitosan make it an ideal agent to react with cross-linking compounds for in situ chemical cross-linking. Other than being non-toxic and biocompatible, chitosan could also be biodegradable when treated by certain enzymes, making it an appropriate candidate for clinical treatment.

The amino group and primary and secondary hydroxyl groups make up the three functional groups of chitosan. Due to the amino group and primary alcohol function of chitosan, a variety of derivatives can be synthesized, such as N-modified chitosan, O-modified chitosan and N, O-modified chitosan. Chitosan derivatives are commonly synthesized to improve their chemical properties, for example, both N-alkyl and N-benzyl derivatives can increase the antimicrobial activity of chitosan, while the phosphorylated derivatives enhance its solubility [21]. 

### 3.1. Preparation of ChNPs

There are various ways to produce NPs, including bottom-up, top-down, and a combination of the two techniques [22]. The “bottom-up” approaches include the reverse micelle medium or microemulsion methods, as well as the polymerization of Ch with methacrylic acid (PMAA) to make Ch-PMAA NPs [23]. Several “top-down” methods are also applied to the synthesis of these nanomaterials, such as milling, high-pressure homogenization, and ultra-sonication [24].

#### 3.1.1. Ionotropic Gelation Method

Currently, ChNPs are synthesized using a bottom-up ionic gelation method in which an anionic cross-linker solution is applied. Sodium tripolyphosphate (TPP), for example, is a negatively charged polyanion that can interact with the positively charged amine group of Ch through electrostatic interaction [25,26,27,28]. There are several anionic cross-linkers such as glutaraldehyde that can be used to synthesize ChNP; however, TPP is more suitable due to its biocompatibility and biodegradability [29]. The chitosan molecular weight (MW), initial concentrations of TPP and chitosan, the chitosan to TPP mass ratio, the degree of acetylation of chitosan, and pH of the reaction media could determine the NP size, hydrodynamic diameter, shape, and monodispersity [27].

#### 3.1.2. Microemulsion Method

Using the microemulsion method, Banerjee et al. [30] produced ChNPs through entrapping chitosan in the aqueous core of the reverse micellar system, followed by cross-linking with glutaraldehyde. The particle size can be controlled by varying the amount of glutaraldehyde.

#### 3.1.3. Polyelectrolyte Complex (PEC) Method

The formulation of PEC takes place as a result of electrostatic interaction between anions and cations, followed by neutralization of charge. The sizes of nanocomplexes formulated can vary from 50 to 700 nm [31]. PH, MW, and concentration were associated with the size and yield of nanoparticles. There was a stronger complexation of nanoparticles at lower pH and moderate MW [32].

#### 3.1.4. Complex Coacervation Method

ChNPs were prepared using a complex coacervation method that combines cationic chitosan with anionic polymers to form coacervates [33]. The NP’s size, surface charge, entrapment efficiency, and release characteristics depend largely on the weight ratio of the two polymers [34]. ChNPs produced by complex coacervation have been reported to improve their stability, biodegradability, photostability, oligomerization, and controlled release of bioactive compounds [35].

#### 3.1.5. Solvent Evaporation Method

The first stage of this method is adding the chitosan solution into the aqueous phase to form an emulsion. Then, the polymer solvent evaporates, resulting in the precipitation of nanospheres. After adding chitosan to ethanol, a pDNA-Tris buffer is also added into the solution with rapid pouring of ethanol under magnetic stirring. Eventually, the NPs are produced by removing the solvent with reduced pressure [36].

#### 3.1.6. Coprecipitation Method

This method is based on the coprecipitation of a chitosan solution (prepared in a low pH acetic acid solution) by the addition of a high pH solution such as ammonium hydroxide, resulting in the formation of chitosan nanoparticles. A coprecipitation of lacticacid–grafted Ch and ammonium hydroxide was used to synthesize highly monodisperse ChNPs and magnetic NPs, which exhibited considerable drug-loading abilities and sustained drug release [37].

#### 3.1.7. Incorporation and Incubation Method

ChNPs can be prepared using this method for the delivery of protein molecules. As part of the incorporation procedure, the protein is premixed with a chitosan solution at a certain pH and temperature [38]. The Ch-protein nanoparticles form spontaneously after the mixing the TPP and the solution. On the contrary, in the incubation method, ChNPs are produced first via TPP coacervation. Then, a protein containing the solution is mixed with the ChNPs. By using this method, the protein is only loaded via adsorption onto nanoparticles’ surfaces [39]. This method produces nanoparticles with a size of about 100–150 nm.

**Figure 2:**
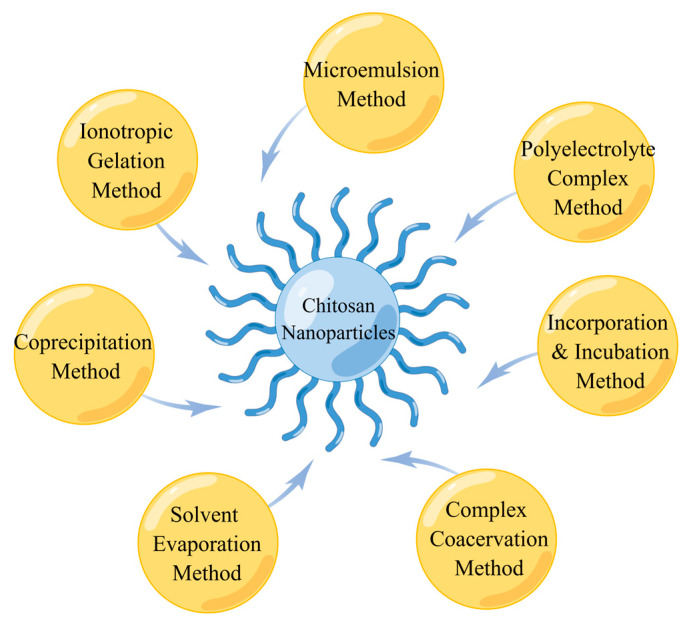
Methods of preparation of chitosan nanoparticles.

In brief, there are several methods of preparing ChNPs including ionotropic gelation, microemulsion, polyelectrolyte complex (PEC), complex coacervation, solvent evaporation, coprecipitation, and incorporation and incubation methods, among which the ionotropic gelation method is most widely applied, as shown in Figure 2.

### 3.2. Characterization of ChNPs 

In order to understand the optimal formation of nanoparticles and their influence on different applications, it is essential to characterize nanomaterials that have been synthesized. Considering the pH value of the standard positive urine is faintly acidic, the ideal IDD system needs to be soluble in weak acidic pH regions. Zeta potential predicts the potential stability of NPs and as the zeta potential increases, so does the stability. The size of NPs is critical when considering their applications in biomedical and pharmaceutical industries. For instance, a smaller ChNP is capable of penetrating capillaries and tissue sinusoids, which can be useful for drug delivery because it has better antibacterial activity and cell penetration than a larger NP [6,7]. In practice, measuring the size of a ChNP is usually difficult due to its polydispersity. For determining the size of nanoparticles, scanning electron microscopy (SEM), transmission electron microscopy (TEM), and atomic force microscopy (AFM) are used in conjunction with dynamic light scattering (DLS) [40,41]. In comparison with DLS, AFM and TEM provide both qualitative and quantitative information on particle sizes, surface morphologies, and particle shapes. However, the quantitative use of SEM or AFM for measurements of particle size is debatable as the particles captured in the images is subjectively determined, which will eventually reduce the reproducibility of these methods for particle size measurements. Additionally, structural changes in ChNP formation have been observed using TEMs, AFMs, SEMs, Fourier-transform infrared spectroscopy (FTIR), and X-ray powder diffraction (XRD) [42,43,44].

### 3.3. Modification of ChNPs 

Mucoadhesive polymers are generally associated with long-term residence times. However, there are studies that report limited or insufficient adhesion of these polymers with chitosan derivates produced with a non-covalent bond [45]. Additionally, there are several methods to modify ChNPs.

#### 3.3.1. Thiolation

Thiomers (thiolated polymers) form covalent bonds with cysteine domains in mucus in order to form mucoadhesive polymers. Mucoadhesion is strengthened by the disulfide bond between thiolated polymers and mucus. Chitosan-based formulations are expected to improve retention mucoadhesion through disulfide bonds. Thiolated chitosans, derived from chitosan, further increase mucoadhesive properties through disulphide bonds with cystein-rich mucus glycoprotein domains. Moreover, their mucosal permeation enhancing properties are enhanced by the regeneration of gluthation (GSH). Furthermore, their ability to bind divalent cations, such as Zn^2+^ or Mg^2+^, which are cofactors of many proteases, makes them potent antiprotease agents. Due to these characteristics, thiolated chitosan is an excellent mucosal delivery system for proteins and peptides [46,47,48].

A system was developed by Barthelmes et al. [49] in which chitosan was modified with thioglycolic acid (TGA) and then fabricated into nanoparticles (CH-TGA). Compared to intact chitosan, the thiomers had superior mucoadhesive properties, allowing prolonged stay on the mucosal surface. An incubation chamber was used to evaluate the NPs’ mucoadhesion in porcine urinary bladders; a 14-fold higher mucoadhesion was achieved using CH-TGA nanoparticles than compared to unmodified chitosan nanoparticles, demonstrating CH-TGA’s superior efficacy. In a recent study, Şenyigit et al. formulated CH-TGA nanoparticles and dispersed them in either chitosan gel (CH-TGA-CH) or poloxamer gel (CH-TGA-P). By using bovine bladder mucosa as a texture analysis, it was determined that the detachment forces for CH-TGA-NP-CH was 1.003 ± 0.048 N while the CH-TGA-NP-P is 0.370 + 0.022 N. Researchers also found that CH-TGA-NP-CH showed better drug permeation than CH-TGA-NP-P in an ex vivo study using a Franz-type diffusion cell.

#### 3.3.2. Boronation

The mucoadhesive properties of boronated chitosan were improved by reaction with 4-carboxyphenylboronic acid, according to Kolawole et al. [50]. Three products with varying degrees of boronate conjugation were synthesized and characterized using ^1^H NMR, FT-IR, and UV–Vis spectroscopy. These polymers were investigated for their ability to in-crease the residence time of the loaded drug in the bladder. Chitosan modification was assessed by ^1^H NMR and ninhydrin testing. Fluorescein sodium was used as a model drug to assess mucoadhesive properties in porcine bladder mucosal tissue using flow-through technique and fluorescent microscopy. Additionally, tensile tests were applied to study the mucoadhesive properties of these polymers on porcine bladder mucosa. Ac-cording to the flow-through and tensile techniques, there was a good correlation in the mucoadhesive profiles of the polymers. Mucoadhesion was significantly affected by the degree of chitosan modification, with greater mucoadhesion observed with higher boronation levels. This implies that the boronated chitosan derivatives could play an important role in improving bladder therapy as an intravesical drug delivery system.

#### 3.3.3. Methacrylation

Kolawole et al. [51] compared the pH-dependent solubility, mucoadhesive properties, and safety profile of unmodified and methacrylated chitosan. In this study, a methacrylated derivative of chitosan was synthesized through chemical modification using methacrylic anhydride to improve its mucoadhesive properties. ^1^H NMR spectroscopy, FT-IR spectroscopy, and UV–Vis spectroscopy were used to characterize the reaction products. Additionally, a ninhydrin test was used to quantify the degree of methacrylation. According to the turbidimetric analysis of pH effects on the aqueous solubility of polymers, the highly methacrylated derivative remained turbid at pH 3–9 and maintained its turbidity. However, solutions of native chitosan and its derivative with low methacrylation remained transparent at pH 6.5 and exhibited a rapid increase in turbidity at pH > 6.5. Based on both in vitro and in vivo studies, the methacrylated derivatives were observed to be more effective than the parent chitosan at retaining fluorescein sodium on bladder mucosa. No significant differences were observed in toxicity between chitosan and its methacrylated derivatives in toxicological studies using MTT assays with UMUC3 bladder cells. This suggests that methacrylated chitosan can be used as a simple and viable synthetic approach to increase mucoadhesive properties of drug carriers. Various approaches to modify ChNPs are listed in Table 1.

## 4. Chitosan in Intravesical Drug Delivery

### 4.1. Bacillus Calmette–Guerin (BCG)

Instillation of BCG, a live attenuated Mycobacterium from the same family as tuberculosis, causes a local inflammatory response in the bladder. Erdogar et al. [52] developed BCG-loaded chitosan nanoparticles to overcome the systemic side effects caused by BCG. Using an ionotropic gelation technique based on the interaction between oppositely charged groups of chitosan and sodium tripolyphosphate (TPP), BCG-loaded chitosan nanoparticles were prepared spontaneously. The particle size was 375 ± 6 nm, zeta potential was +17.7 ± 0.7 mV, and the encapsulation efficiency was about 42%. These nanoparticles showed improved antitumor efficacy, increased survival rate, and reduced side effects arising from systemic uptake of the locally applied non-encapsulated BCG.

### 4.2. Mitomycin C (MMC)

Antitumor antibiotic MMC has therapeutic activity against a variety of human tumors and is a preferred treatment for superficial bladder tumors in intravenous chemo-therapy. In acidic environments, MMC rapidly degrades and causes dose-dependent allergic reactions upon systemic uptake, as well as chemical cystitis. Erdogar et al. [53] evaluated the cationic core-shell nanoparticles’ formulations (based on chitosan (CS) and poly-e-caprolactone (PCL)) for their antitumor efficacy after intravesical administration in a bladder tumor-induced rat model. As illustrated by the Kaplan–Meier curve in this study, chitosan-coated poly-e-caprolactone (CS-PCL) nanoparticles demonstrated the longest survival rate. In addition, no MMC was detected in the blood after intravenous instillation, suggesting that there was no systemic uptake. The CS-PCL nanoparticle size was 319 ± 5 nm, zeta potential was +10.5 ± 1 mV, and the encapsulation efficiency was about 35%.

### 4.3. Gemcitabine

In addition to its antitumor properties, gemcitabine hydrochloride (Gem-HCl) is a deoxycytidine analog with a wide therapeutic range. Gem-HCl is effective and well tolerated when used systemically for a number of tumors, including superficial bladder cancer. Currently, Gem-HCl appears to be a promising new candidate for standard intravesical therapy. When compared with other intravesical drugs such as doxorubicin, epirubicin, mitomycin-C, or thiotepa, Gem-HCl showed greater activity. In order to prolong the drug’s intravesical residence time, to obtain controlled drug release, and to reduce drug elimination via periodic urination, Ay Şenyiğit et al. [54] developed an IDD system of gemcitabine HCl-loaded chitosan–thioglycolic acid nanoparticles. They successfully developed chitosan-thioglycolic acid nanoparticles via ionotropic gelation that reached an encapsulation efficiency of nearly 20%. The NPs had a mean diameter of 174.5 ± 3.762 nm and zeta potential of 32.100 ± 0.575 mV. Both in vitro and ex vivo characterization studies showed that nanoparticle-loaded chitosan can prolong the residence time in the bladder and improve treatment efficacy of Gem-HCl, thus making it a promising carrier to deliver Gem-HCl intravesically.

### 4.4. Paclitaxel (PTX)

PTX is widely used as an effective first-line treatment for various cancers, including bladder cancer [55]. Yongjia Liu et al. [56] provided paclitaxel/chitosan (PTX/CS) nanosupensions (NSs) with sustained and prolonged delivery of paclitaxel with enhanced therapeutic efficiency in intravesical bladder cancer. The PTX/CS NSs preparation was based on the PTX and CS molecular self-assembly and ultrasonic cutting. It was found that PTX/CS NSs exhibited rod-shaped shapes with mean diameters around 200 nm. Water disperses them well in the absence of protective agents, and their positive charge enable their easy adsorption on the bladder’s mucosa via electrostatic attraction. The PTX/CS NSs have also been shown to have a high drug-loading capacity and paclitaxel release that can last for ten days or more. Experimental studies in both cells and animals showed that PTX/CS NSs were safe and effective against tumors. In the case of bladder cancer, the PTX/CS NSs may act as an intravesical anti-cancer nanomedicine.

### 4.5. Cisplatin

In clinical studies, doxorubicin (Dox), cisplatin (CDDP) and cisplatin analogs are widely used highly water-soluble anti-cancer drugs [57]. Considering intravesical chemotherapy of NMIBCs, the CDDP-based treatment has proven to be less effective than other drugs [58]. Possibly, the small, neutral CDDP molecule is poorly adsorbed and has low penetration into the bladder urothelium as a result of its poor adsorptive properties. As a potential solution to overcome adverse side effects and drug resistance, synergism offers the advantage of increased therapeutic efficacy. Recently, the co-instillation of two or more drugs showed the potential for intravesical chemotherapy against NMIBCs [59]. Lu et al. [60] provided a “two-in-one” combination of Dox and peptide-modified cisplatin (Pt–ALy) loaded in positively charged mucoadhesive chitosan–polymethacrylic acid (CM) nanocapsules as a therapeutic material against UMUC3 bladder cancer cells. According to dynamic light scattering, the size of these CM nanocapsules was estimated to be 114.4 ± 11.8 nm. Additionally, the zeta potential of these CM nanocapsules was about +15 mV. There is no evidence of urothelial damage in the bladder when CM nanocapsules are used on the bladder’s luminal surface, making it an ideal strategy for long-term drug retention in the bladder and effective delivery to the urothelium. With the sustained drug delivery property and significant synergistic effects of the CM–Dox–PtALy nanocapsule, it is possible to reduce the required dosage of each drug, and therefore it is becoming a promising IDD system against NMIBCs.

### 4.6. Genetic Agents

As NPs are internalized into cells via endocytosis, ChNPs are able to deliver biologically active materials to cells without compromising their integrity [61]. The delivery of biopharmaceuticals requires innocuous delivery systems capable of protecting sensitive biologics against enzymatic and chemical degradation [62,63]. It is reported that the Ch–DNA complexes of 50–100 nm in size could be effectively transfected into HeLa cells within an hour of exposure without associated cellular toxicity at the concentrations of 100 μg/mL. Meanwhile, cytotoxicity was observed in the control polyethylenimine–DNA complexes dosed at the same concentrations [64]. It is possible for ChNPs to interact with negatively charged DNA and form polyelectrolytes during gene delivery. When DNA was included in these complexes, nuclease degradation was ineffective, resulting in more efficient transfection [65].

#### 4.6.1. siRNA

Survivin, an apoptosis inhibitor, is highly expressed in bladder cancer cells. In a study by Martin et al. [66], a positively charged mucoadhesive chitosan with low molecular weight (2.5 or 20 kDa) was applied to increase the transurothelial migration and tumor cell uptake of poly (lactic-co-glycolic acid; PLGA)-nanoparticles (NP) through surface modification. Compared to unmodified nanoparticles, chitosan-modified nanoparticles showed better uptake in vivo, and the nanoparticles modified using the chitosan with the higher molecular weight showed greater uptake. Additionally, ex vivo studies utilizing human urothelium continued to show similar results to in vivo studies, proving chitosan’s superior ability to enhance uptake. In addition to being able to transport large quantities of siRNA across the urothelium and/or to the tumor site, chitosan with low molecular weight has the ability to increase therapeutic response. It was demonstrated for the first time that PLGA NPs, functionalized with siRNA (and unmodified siRNA PLGA NPs) packaged in chitosan with low molecular weight improved binding in both human ex vivo samples and mouse in vivo samples. Furthermore, prolonged survivin knockdown and reduced tumor growth was observed while using the low molecular weight Ch 2.5-modified PLGA NPs encapsulating siRNA.

CD44 is highly expressed in an amount of tumor cells, and plays important roles in a variety of cellular functions, including cancer cell growth, migration, metastasis, and resistance to apoptosis [67]. Ye Liang et al. [68] developed self-cross-linkable chitosan-hyaluronic acid dialdehyde nanoparticles for CD44-targeted siRNA delivery to treat bladder cancer. They used the ionotropic gelation method to fabricate nanoparticles. SiRNA-loaded nanoparticles were mixed with a hyaluronic acid dialdehyde (HAD) solution under ultrasound to produce siRNA-loaded targeting nanoparticles (siRNA@CS-HAD NPs). The siRNA@CS-HAD NPs had a 100–120 nm size and good stability. High siRNA encapsulation capability, low cytotoxicity, and good blood compatibility of the nanoparticles was observed in both in vitro and in vivo experiments. These results indicated that siRNA@CS-HAD NPs could be a promising treatment method for the targeted therapy of bladder cancers with high CD44 expression.

#### 4.6.2. miRNA

RNA interference techniques represent a promising approach in therapeutics. While the small-interfering-RNA-based approaches have been widely studied and translated into clinical investigations, microRNA-based approaches are also attractive, owing to their “one hit, multiple targets” concept [69]. However, there is no research available for the intravesical application of miRNA ChNPs. Table 2 provides examples of where chitosan and its derivatives are utilized in intravesical drug delivery.

## 5. Conclusions

Although chitosan can temporarily abolish urothelium’s barrier function, which can allow drugs to penetrate deeper into the bladder layer, it offers a wide range of biomedical applications in the urinary bladder epithelium. In conjunction with cytostatic and chemotherapy, chitosan can be a very effective and safe supplementary system in treating bladder tumors. In addition to their local topical application, ChNPs have the benefit of avoiding the systemic side effects associated with drugs previously administered orally or intravenously. 

In order to prevent systemic absorption of the drugs, biodistribution and pharmaco-kinetic studies should also be conducted. In spite of promising outcomes, many ongoing clinical trials for these IDD systems remain unsuccessful. The cytotoxicity of ChNPs has been demonstrated both in vitro and in vivo in several studies. Currently, we have insufficient knowledge of Ch-based nanomaterials, and there is an urgent need to conduct further investigation into their fabrication and biological properties. In addition, rodent models have been used in most of the in vivo studies to date; therefore, studies in large animal models are required to establish whether these novel drugs would be clinically acceptable. Nevertheless, despite a few possible shortcomings, ChNPs still have a bright future in intravesical bladder cancer treatment as well as pharmaceutical nanotechnology.

## Figures and Tables

**Table 1:** Description of methods to modify chitosan nanoparticles.

Methods	Chemical	Merits	References
Thiolation	thioglycolic acid (TGA)	increase mucoadhesive properties	[49]
Boronation	4-carboxyphenylboronic acid	increase mucoadhesive properties	[50]
Methacrylation	methacrylic anhydride	increase mucoadhesive properties	[51]

**Table 2:** Chitosan in intravesical drug delivery.

Drugs	Chitosan Derivatives	Methods to Prepare Nanoparticles	Results	Ref.
Bacillus Calmette—Guerin (BCG)	BCG-loaded chitosan nanoparticles	ionotropic gelation technique	BCG-loaded chitosan nanoparticles resulted in significantly longer survival than BCG commercial product in rat model (up to 86 days of survival with no systemic side effects)	[52]
Mitomycin C (MMC)	MMC-loaded chitosan nanoparticles	ionotropic gelation technique	nanoparticle formulations have longer retention times in the bladder resulting from stronger mucosal and cellular interactions	[53]
Gemcitabine	gemcitabine HCl-loaded chitosan–thioglycolic acid nanoparticles	ionotropic gelation of chitosan–TGA with TPP	Both in vitro/ex vivo characterization studies revealed that nanoparticle-loaded chitosan could be used as an alternative carrier for intravesical administration of gemcitabine HCl, thereby prolonging its residence time in the bladder and improving treatment efficacy	[54]
Paclitaxel (PTX)	paclitaxel/chitosan (PTX/CS) nanosupensions (NSs)	sonication	The PTX/CS NSs could provide sustained chemotherapeutic agent delivery with significant anti-cancer efficacy against intravesical bladder cancer (verified both in vitro and in situ bladder cancer model)	[56]
Cisplatin & Doxorubicin(Dox)	chitosan–polymethacrylic acid (CM) nanocapsules	electrostatic interactions between chitosan and methacrylic acid (MAA) chains undergoing polymerization	The CM–Dox–PtALy nanocapsule showed sustained drug delivery property and significant synergistic effects in ex vivo study	[60]
survivin siRNA	chitosan-modified NPs encapsulating survivin siRNA	double emulsion solvent evaporation technique	NPs functionalized with low molecular weight chitosan encapsulating siRNA could improve binding in both human ex vivo specimens and in mouse in vivo specimens, and also prolong the survivin knockdown and reduced tumor growth	[66]
Bcl2(CD44)-cy3-siRNA	chitosan-modified NPs encapsulating survivin siRNA	ionotropic gelation technique	The siRNA@CS-HAD NPs had high siRNA encapsulation capability, low cytotoxicity and good blood compatibility. In vivo study showed that this NP can specifically accumulate at the tumor site and had strong effect on inhibiting the targeted oncogene and inhibit the tumor growth.	[68]

TGA: thioglycolic acid.

## Data Availability

Not applicable.
